# Clinical and Microbiological Characteristics of a Community-Acquired Carbapenem-Resistant *Escherichia coli* ST410 Isolate Harbouring *bla*NDM-5-Encoding IncX3-Type Plasmid From Blood

**DOI:** 10.3389/fmed.2021.658058

**Published:** 2021-06-11

**Authors:** Ji-Na Gu, Lin Chen, Xing-Bei Weng, Xiao-Yan Yang, Dan-Mei Pan

**Affiliations:** ^1^Department of Infectious Medicine, Hwa Mei Hospital, University of Chinese Academy of Sciences, Ningbo, China; ^2^Department of Laboratory Medicine, Ningbo First Hospital, Ningbo, China

**Keywords:** whole genome sequence, *Escherichia coli* ST410, NDM-5, IncX3-type plasmid, high-risk clone

## Abstract

**Objectives:** The aim of this research was to investigate the clinical and microbiological characteristics of a case of community-acquired carbapenem-resistant *Escherichia coli* isolated from a patient with a bloodstream infection in China.

**Methods:** *Escherichia coli* Huamei202001 was recovered from the first blood culture from a patient hospitalised in China. An antimicrobial susceptibility test was performed, and the genome was sequenced on an Illumina HiSeq X 10 platform with a 150-bp paired-end approach. The generated sequence reads were assembled using Unicycler, and the whole genome sequence data were analysed using bioinformatics tools. Moreover, the patient and her main family members obtained a faecal sample screening test for CRE, the positive strain was further isolated and the identification and antimicrobial susceptibility testing was performed.

**Results:** *Escherichia coli* Huamei202001 belonged to sequence type 410. In addition, a *bla*NDM-5-encoding IncX3-type plasmid was responsible for the spreading of carbapenem resistance. Only the patient was detected as having a positive faecal sample screening test for CRE. Strain Fec01 was identified as *E. coli*, and the antibiotic susceptibility profile was the same as that of *E. coli* Huamei202001.

**Conclusions:** *Escherichia coli* Huamei202001 is defined as community-acquired carbapenem-resistant Enterobacteriaceae. The clone ST410 that harbours the *bla*NDM-5-encoding IncX3-type plasmid is causing new high-risk clones globally. Thus, infection control measures should be strengthened to curb the dissemination of IncX3.

## Introduction

The emergence and spread of carbapenem-resistant Enterobacteriaceae (CRE) has created an escalating global threat with the dissemination of carbapenemase genes. The most common carbapenemase genes include *bla*KPC, *bla*NDM, *bla*VIM, *bla*IMP, and *bla*OXA-48-like (1). A nationwide survey conducted in China showed that acquisition of two carbapenemase genes, *bla*KPC-2 and *bla*NDM, was responsible for phenotypic resistance in 90% of the CRE strains tested (58 and 32%, respectively) (2). The incidence of CRE occurring in either community-associated or community-onset patients ranges from 0.04 to 29.5% worldwide; therefore, the presence of CRE in the community poses an urgent public health threat (3). NDM-5-producing ST167 (4), ST290 (5), ST361 (6), and ST410 (7, 8) *Escherichia coli* have been reported. The *blaN*DM-5-encoding IncX3-type plasmid is responsible for disseminating carbapenem-resistant *E. coli* ST410, which has developed into a new high-risk clone globally.

According to the Chinese XDR Consensus, the possible therapeutic options for treating CRE infection are narrow, and the most frequently used antimicrobials used for combination therapies include aminoglycosides, carbapenems, colistin, fosfomycin, and tigecycline (9). The mortality rate of bloodstream CRE infections is close to 70% (10), but the clinical and microbiological characteristics of *E. coli* ST410 have not been thoroughly elucidated. This research describes the successful cure of a case of community-acquired bloodstream CRE infection, aiming to investigate the clinical and microbiological characteristics to provide evidence for the clinical control of CRE.

## Materials and Methods

### Isolation and Identification

*Escherichia coli* Huamei202001 was recovered from the first blood culture collected on January 7, 2020, from a 59-year-old female patient hospitalised at Hwa Mei Hospital, University of Chinese Academy of Sciences, Zhejiang Province, China. The study was reviewed and approved by the Ethics Committee of Hwa Mei Hospital, University of Chinese Academy of Sciences (Approval no. PJ-NBEY-KY-2021-015-01). On January 3, 2020, this patient suffered from fever, and the highest temperature was 39.7°C, with chills, nausea and vomiting once. Two days later, she visited Hwa Mei Hospital and was administered levofloxacin (0.5 g ivgtt, qd) for 2 days with a white blood cell count of 6.0 × 10^9^/L, neutrophil% of 90.0%, haemoglobin 128 g/L, platelets 116 × 10^9^/L and C-reactive protein (CRP) 73.0 mg/L. On January 7, the symptoms continued, and the patient was hospitalised (day 0) in the infectious disease ward and was diagnosed with sepsis, hypertension and hepatic cysts, with a white blood cell count of 11.3 × 10^9^/L, neutrophil% of 90.3%, haemoglobin 127 g/L, platelets 60 × 10^9^/L, CRP 250.0 mg/L and procalcitonin 40.37 ng/ml (Supplementary Table 1). Blood culture was collected, and chest CT and abdominal CT showed some chronic inflammation in both lungs and multiple low-density masses in the liver; thus, one liver cyst with infection was considered. Empiric therapy was administered with imipenem (1.0 g q8 h). On day 2, ultrasound-guided percutaneous puncture of the infected liver cyst was performed. A total of 45 ml yellow liquid was extracted. The puncture fluid showed a white blood cell count of 1.879 × 10^9^/L and neutrophil% of 85.0%. However, the puncture fluid culture was negative. Because of the blood culture indicating carbapenem-resistant *E. coli*, tigecycline (100 mg q12 h) and polymyxin B (750,000 U q12 h, 1,000,000 U first dose) were administered. On day 9, the patient complained of numbness of the extremities, polymyxin neurotoxicity was considered and the dose of polymyxin B was reduced to 500,000 U q12 h, with the white blood cell count being 12 × 10^9^/L, CRP 49.47 mg/L and procalcitonin 0.28 ng/ml. On day 13, the patient's creatinine increased progressively, and polymyxin nephrotoxicity was considered, so polymyxin was discontinued, with a white blood cell count of 6.4 × 10^9^/L and a CRP level of 16.14 mg/L. On day 18, as a result of the antimicrobial susceptibility testing showing that the strain was susceptible to fosfomycin, administration was changed to tigecycline (50 mg q12 h) and fosfomycin (12 g q12 h) with a white blood cell count of 5.1 × 10^9^/L, a CRP level of 7.45 mg/L and a procalcitonin level of 0.95 ng/ml. On day 24, all antibiotics were discontinued because of the normal laboratory findings, and the patient's condition was closely observed. On day 29, the patient was discharged. *Escherichia coli* Huamei202001 was identified with a VITEK 2 compact automated microbiology system (bioMerieux, Marcy-l'Etoile, France).

### Antimicrobial Susceptibility Testing

Antimicrobial susceptibility testing of MICs was performed by a VITEK 2 compact automated microbiology system, and testing of fosfomycin was performed by the K-B method (Oxoid, Basingstoke, UK), with susceptibility defined according to the Clinical and Laboratory Standards Institute (CLSI) (M100-S30). Testing of tigecycline was performed by the broth microdilution MIC determination method, and the broth was prepared fresh on the day of use (Oxoid), with susceptibility defined according to the European Committee on Antimicrobial Susceptibility Testing (EUCAST) (version 11.0, for tigecycline) guidelines.

### Genome Sequencing and Bioinformatics Analysis

The strain was sent to Zhejiang Tianke Hi-Tech Development Co., Ltd. (Tianke, Hangzhou, China) for genome sequencing. Genomic DNA was extracted using a plant Genomic DNA kit (DP305, Tiangen, Beijing, China). The library was sequenced on an Illumina HiSeq X Ten platform (Illumina Inc., San Diego, CA, USA), and 150 bp paired-end reads were generated at a depth of 250×. The raw reads of *E. coli* Huamei202001 were assembled into draught genomes using Unicycler. The contigs were annotated by Rapid Annotation using Subsystem Technology, and whole genome sequence data analyses were performed using these bioinformatics tools (i.e., ResFinder v.3.2, MLST v.2.0, ISfinder, Virulence Finder v.2.0, Plasmid Finder v.2.0, pMLST v.2.0 and FimTyper v.1.0) available from the Centre for Genomic Epidemiology (http://www.genomicepidemiology.org/).

### Faecal Sample Screening Test for CRE

To trace whether the source of *E. coli* Huamei202001 originated from the intestine, a faecal sample screening test for CRE was performed. Faecal samples were collected from the patient and her main family members and then inoculated on CRE screening plates (11). Colonies on CRE screening plates were further isolated, and identification and antimicrobial susceptibility testing were performed as noted above.

### Pulsed-Field Gel Electrophoresis

Genomic DNA was prepared as described previously (12). Isolated colonies were harvested from Mueller–Hinton agar plates after overnight incubation at 37°C, and the suspension was adjusted to a concentration of 10^9^ CFU/ml in cell suspension buffer (100 mM Tris–HCl, 100 mM EDTA, pH = 8). After a short incubation of ~5–10 min at 37°C, the bacterial suspension was mixed with an equal volume of 1% Gold Agarose (Lonza, Rockland, MD, USA) and allowed to solidify in a 100-μl plug mould. The DNA block was incubated overnight at 54°C in 1 ml of cell lysis buffer (50 mM Tris–HCl, 50 mM EDTA, 1% sarcosyl, 100 μg/ml proteinase K, pH = 8). To eliminate the lysed bacterial material and inactivate proteinase K activity, the DNA blocks were washed four times at 50°C in 4 ml of Tris–EDTA buffer (100 mM Tris–HCl, 1 mM EDTA, pH = 8). A slice of each plug was cut and incubated with *Xba*I (Takara, Shiga, Japan). Restriction fragments of DNA were separated by pulsed-field gel electrophoresis (PFGE) with a CHEF Mapper apparatus (Bio-Rad, Hercules, CA, USA) through 1% Gold Agarose. Electrophoresis was performed at 6 V/cm and 14°C. The run time was 20 h, with the pulse time ramping from 5 to 35 s. *Xba*I-digested DNA of *Salmonella enterica* serotype *Braenderup* H9812 was electrophoresed as the size marker.

## Results

Antibiotic susceptibility profiles showed that *E. coli* Huamei202001 was resistant to most antimicrobial agents, including ampicillin, amoxicillin/clavulanic acid, cefazolin, cefoxitin, ceftriaxone, cefepime, aztreonam, piperacillin/tazobactam, imipenem, ertapenem, ciprofloxacin, levofloxacin and trimethoprim-sulfamethoxazole, but remained susceptible to gentamicin, amikacin, nitrofurantoin and tigecycline.

Acquired resistance genes to aminoglycosides [*aph*(3′)-Ia, *aad*A1, *aad*A2,ant(3″)-IIa, *aac*(6′)-Ib10, and *aac*(6′)-Ib-*cr*], beta-lactams (*bla*CTX-M-14 and *bla*NDM-5), fluoroquinolones [*aac*(6′)-Ib-*cr*], trimethoprim (*dfr*A12), sulfonamides (*sul*2), tetracyclines (*tet*M), macrolides [*mdf* (A)], phenicol (*cml*A1, *flo*R) and disinfectants (*qac*H) were identified. In addition, analysis of quinolone resistance-determining regions of the *gyrA* and *parC* genes revealed the presence of multiple and diverse mutations in *gyr*A (S83 L and D87N) and *par*C (S80I). Mutations in the *gyr*A and *par*C genes have been reported as major mechanisms of fluoroquinolone/quinolone resistance involved in DNA gyrase and topoisomerase IV alterations and are often associated with high-level quinolone resistance in Enterobacteriaceae.

Analysis of the draught genome sequences demonstrated that carbapenem-resistant *E. coli* Huamei202001 belonged to ST410, whereas identification of plasmid replicons revealed that it carried a *bla*NDM-5-encoding IncX3-type plasmid and IncFIA, IncFIB and IncI1 plasmids.

The strain harboured virulence genes, including siderophore receptor (*fyu*A), ferric aerobactin receptor (*iut*A), iron transport protein (*sit*A), tellurium ion resistance protein (*ter*C), aerobactin synthetase (*iuc*C), afimbrial adhesion (*afa*ABCDE) and long polar fimbriae (*lpf* A), suggesting that the strain may have significant pathogenic potential.

**Figure 1:**
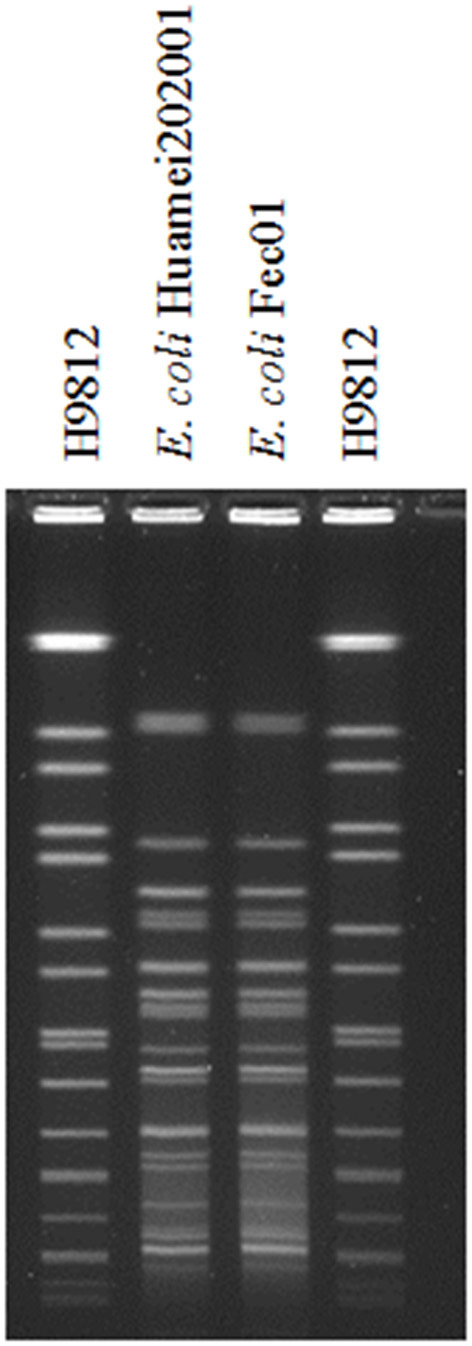
PFGE fingerprinting between strain *E. coli* Huamei202001 and strain *E. coli* Fec01, H9812: *Salmonella* H9812 molecular marker.

Only the patient was detected as having a positive faecal sample screening test for CRE. Isolation Fec01 was identified as *E. coli*, and the antibiotic susceptibility profile was the same as that of *E. coli* Huamei202001. Moreover, PFGE fingerprinting between strain Fec01 and strain Huamei202001 was highly similar (Figure 1). However, her main family members all showed negative results.

## Discussion

Patients who were hospitalised in the 2 weeks before admission or transferred from other hospitals are defined as having hospital-acquired infections (13). A positive culture taken ≤48 h after admission could be classified as healthcare-associated strain with additional criteria (14). A positive culture that did not meet the criteria above was considered to be a strictly community-acquired infection (14–16). Moreover, investigations on CRE have mainly focused on nosocomial infections, and only a few community-acquired CRE infections have been documented (3). Therefore, according to the definition, *E. col*i Huamei202001 is a community-acquired CRE that belongs to ST410 and carries a *bla*NDM-5-encoding IncX3-type plasmid, which is causing new international high-risk clones (17) not only in hospitals in eastern China (7, 8), Egypt (18) and Denmark (17) but also in domestic animals in China (19) and South Korea (20, 21); such risk is even found in the environment, such as in rivers in Switzerland (9) and sewage in northeast India (22). These investigations show that IncX3 is a key element in disseminating *bla*NDM-5 among *E. coli*, even among various species. Thus, infection control measures should be strengthened to curb the spread of highly transferable plasmid-borne carbapenemases.

We infer that bloodstream infection of Huamei202001 is most likely from intestinal colonisation of Fec01, which is due to highly similar PFGE fingerprinting between strain Fec01 and strain Huamei202001 (Figure 1). Another study also supported the strong association between intestinal colonisation and bloodstream infection (23). However, the origin of strain Fec01 is unclear because of the negative results of the faecal sample screening test for CRE among the patient's main family members.

In addition, from day 0 to day 2, imipenem was administered empirically without antibiotic susceptibility profiles, and it seemed effective because the levels of white blood cells, C-reactive protein, procalcitonin and creatinine all decreased (Supplementary Table 1). Antibiotic susceptibility profiles showed that the MIC of imipenem to *E. coli* Huamei202001 was ≥16 mg/L, so it is difficult to explain the mechanism by which imipenem was effective. After imipenem was discontinued, antibiotic administration was changed to a combination of tigecycline and polymyxin B according to the Chinese consensus statement (24). However, the possibility of polymyxin neurotoxicity was considered (25) for the numbness of the extremities, so polymyxin was discontinued. Because the strain was susceptible to fosfomycin, administration was changed to a combination of tigecycline and fosfomycin until day 24. All laboratory findings showed normal results (Supplementary Table 1), and antibiotic administration was discontinued. Finally, the patient recovered and was discharged, which was due to proper antibiotic administration.

This is a rare case report of the complete occurrence and development process of community-acquired CRE infection in China, including the diagnosis and antibiotic administration process, which was finally successfully cured. This outcome demonstrates the complexity and intelligence of antibiotic administration in the clinic. Furthermore, clone ST410 that harbours a *bla*NDM-5-encoding IncX3-type plasmid was analysed by whole genome sequencing and then was discussed to understand the spread of the clone worldwide.

## Data Availability Statement

The datasets presented in this study can be found in online repositories. The names of the repository/repositories and accession number(s) can be found below: https://www.ncbi.nlm.nih.gov/nuccore/JAENHW000000000.

## Ethics Statement

The studies involving human participants were reviewed and approved by Ethics Committee of Hwa Mei Hospital, University of Chinese Academy of Sciences. The patients/participants provided their written informed consent to participate in this study.

## Author Contributions

All authors listed have made a substantial, direct and intellectual contribution to the work, and approved it for publication.

## Conflict of Interest

The authors declare that the research was conducted in the absence of any commercial or financial relationships that could be construed as a potential conflict of interest.

## References

[1] Weng XB Shi QC Wang S Shi YB Sun DH Yu YS. The characterization of OXA-232 carbapenemase-producing ST437 *Klebsiella pneumoniae* in China. Can J Infect Dis Med Microbiol. (2020) 2020:5626503. 10.1155/2020/5626503 32724486 PMC7366223

[2] Zhang R Liu LZ Zhou HW Chan EW Li JP Fang Y. Nationwide surveillance of clinical carbapenem-resistant Enterobacteriaceae (CRE) strains in China. EBioMedicine. (2017) 19:98–106. 10.1016/j.ebiom.2017.04.032 28479289 PMC5440625

[3] Kellya AM Barun Mathemab ELL Larson EL. Carbapenem- resistant Enterobacteriaceae in the community: a scoping review. Int J Antimicrob Agents. (2017) 50:127–34. 10.1016/j.ijantimicag.2017.03.012 28647532 PMC5726257

[4] Garcia-Fernandez A Villa L Bibbolino G Bressan A Trancassini M Pietropaolo V. Novel insights and features of the NDM-5-producing *Escherichia coli* sequence type 167 high-risk clone. mSphere. (2020) 5:e00269–20. 10.1128/mSphere.00269-20 32350092 PMC7193042

[5] Wang Z Li M Shen X Wang L Liu L Hao Z. Outbreak of bla(NDM-5)-harboring *Klebsiella pneumoniae* ST290 in a Tertiary Hospital in China. Microb Drug Resist. (2019) 25:1443–8. 10.1089/mdr.2019.0046 31334685

[6] Park Y Choi Q Kwon GC Koo SH. Emergence and transmission of New Delhi metallo-beta-lactamase-5-producing *Escherichia coli* sequence type 361 in a Tertiary Hospital in South Korea. J Clin Lab Anal. (2020) 34:e23041. 10.1002/jcla.23041 31541503 PMC7031584

[7] Sun P Xia W Liu G Huang X Tang C Liu C. Characterization of bla (NDM-5)-positive *Escherichia coli* prevalent in a University hospital in Eastern China. Infect Drug Resist. (2019) 12:3029–38. 10.2147/IDR.S225546 31576153 PMC6767761

[8] Bi RR Kong ZY Qian HM Jiang F Kang HQ Gu B. High prevalence of bla NDM variants among carbapenem-resistant *Escherichia coli* in Northern Jiangsu Province, China. Front Microbiol. (2018) 9:2704. 10.3389/fmicb.2018.02704 30483231 PMC6243109

[9] Bleichenbacher S Stevens MJA Zurfluh K Perreten V Endimiani A Stephan R. Environmental dissemination of carbapenemase-producing Enterobacteriaceae in rivers in Switzerland. Environ Pollut. (2020) 265:115081. 10.1016/j.envpol.2020.115081 32806462

[10] Friedman ND Carmeli Y Walton AL Schwaber MJ. Carbapenem-resistant Enterobacteriaceae: a strategic roadmap for infection control-corrigendum. Infect Control Hosp Epidemiol. (2017) 38:1136.3. 10.1017/ice.2017.141 28294079

[11] Adler A Navon-Venezia S Moran-Gilad J Marcos E Schwartz D Carmeli Y. Laboratory and clinical evaluation of screening agar plates for detection of carbapenem-resistant Enterobacteriaceae from surveillance rectal swabs. J Clin Microbiol. (2011) 49:2239–42. 10.1128/JCM.02566-10 21471338 PMC3122751

[12] Xu Q Fu Y Zhao F Jiang Y Yu Y. Molecular characterization of carbapenem-resistant *Serratia marcescens* clinical isolates in a Tertiary Hospital in Hangzhou, China. Infect Drug Resist. (2020) 13:999–1008. 10.2147/IDR.S243197 32308441 PMC7152788

[13] Tang HJ Hsieh CF Chang PC Chen JJ Lin YH Lai CC. Clinical significance of community- and healthcare-acquired carbapenem-resistant Enterobacteriaceae isolates. PLoS ONE. (2016) 11:1–8. 10.1371/journal.pone.0151897 26999356 PMC4801408

[14] Hu HB Mao JC Chen YY Wang J Zhang PP Jiang Y. Clinical and microbiological characteristics of community-onset carbapenem-resistant enterobacteriaceae isolates. Infect Drug Resist. (2020) 13:3131–43. 10.2147/IDR.S260804 32982328 PMC7494230

[15] Kang CI Wi YM Ko KS Chung DR Peck KR Lee NY. Outcomes and risk factors for mortality in community-onset bacteremia caused by extended-spectrum beta-lactamase-producing *Escherichia coli*, with a special emphasis on antimicrobial therapy. Scand J Infect Dis. (2013) 45:519–25. 10.3109/00365548.2013.775479 23509913

[16] Quan J Zhao D Liu L Chen Y Zhou J Jiang Y. High prevalence of ESBL-producing *Escherichia coli* and *Klebsiella pneumoniae* in community-onset bloodstream infections in China. J Antimicrob Chemother. (2017) 72:273–80. 10.1093/jac/dkw372 27624571

[17] Roer L Overballe-Petersen S Hansen F Schønning K Wang M Røder BL. *Escherichia coli* sequence type 410 is causing new international high-risk clones. mSphere. (2018) 3:e00337–18. 10.1128/mSphere.00337-18 30021879 PMC6052333

[18] Soliman AM Zarad HO Nariya H Shimamoto T Shimamoto T. Genetic analysis of carbapenemase-producing Gram-negative bacteria isolated from a University teaching hospital in Egypt. Infect Genet Evol. (2020) 77:104065. 10.1016/j.meegid.2019.104065 31634643

[19] Wang J Xia YB Huang XY Wang Y Lv LC Lin QQ. Emergence of bla(NDM-5) in enterobacteriaceae isolates from companion animals in Guangzhou, China. Microb Drug Resist. (2020). 10.1089/mdr.2020.0210. [Epub ahead of print]. 33216688

[20] Hong JS Song W Jeong SH. Molecular characteristics of NDM-5-producing *Escherichia coli* from a cat and a dog in South Korea. Microb Drug Resist. (2020) 26:1005–8. 10.1089/mdr.2019.0382 32043911

[21] Hong JS Song W Park HM Oh JY Chae JC Han JI. First detection of New Delhi metallo-beta-lactamase-5-producing *Escherichia coli* from companion animals in Korea. Microb Drug Resist. (2019) 25:344–9. 10.1089/mdr.2018.0237 30379599

[22] Paul D Babenko D Toleman MA. Human carriage of cefotaxime-resistant Escherichia coli in North-East India: an analysis of STs and associated resistance mechanisms. J Antimicrob Chemother. (2020) 75:72–6. 10.1093/jac/dkz416 31622465

[23] Kontopoulou K Iosifidis E Antoniadou E Tasioudis P Petinaki E Malli E. The clinical significance of carbapenem-resistant *Klebsiella pneumoniae* rectal colonization in critically ill patients: from colonization to bloodstream infection. J Med Microbiol. (2019) 68:326–35. 10.1099/jmm.0.000921 30688629

[24] Chinese XDR Consensus Working Group Guan X He L Hu B Hu J Huang X. Laboratory diagnosis, clinical management and infection control of the infections caused by extensively drug-resistant Gram-negative bacilli: a Chinese consensus statement. Clin Microbiol Infect. (2016) 22(Suppl. 1):S15–25. 10.1016/j.cmi.2015.11.004 26627340

[25] Kelesidis T Falagas ME. The safety of polymyxin antibiotics. Expert Opin Drug Saf. (2015) 14:1687–701. 10.1517/14740338.2015.1088520 26365594 PMC8082492

