# Supplementary material for: Clinical and Microbiological Characteristics of a Community-Acquired Carbapenem-Resistant Escherichia coli ST410 Isolate Harbouring blaNDM-5-Encoding IncX3-Type Plasmid From Blood
Source: Front Med (Lausanne). 2021 Jun 11;8:658058. doi: 10.3389/fmed.2021.658058 (PMC8226244; doi:10.3389/fmed.2021.658058)
Supplement: Supplementary file 1 [file Table_1.doc]

**Table S1 Laboratory Findings of Bloodstream Infection Patient Caused by Community Acquired Carbapenem-resistant Enterobacteriaceae**

| Date | white blood  cell (10^9^/L) | c-reactive (mg/dl) | procalcitonin (ng/ml) | creatinine  (umol/L) |
| --- | --- | --- | --- | --- |
| January. 3(Day -4)  (fever, 39.7℃) | / | / | / | / |
| January. 5(Day -2)  (outpatient visit) | 6.0 | 73.0 | / | / |
| Jan. 7 (Day 0)(admission) | 11.3 | 328.38 | 40.37 | 114.7 |
| Jan.9 (Day 2) | 7.3 | 136.61 | 4.85 | 38.7 |
| Jan.11 (Day 4) | 9.7 | 84.88 | 0.70 | 39.4 |
| Jan.13 (Day 6) | 10.1 | 103.85 | 0.31 | 49.9 |
| Jan.15 (Day 8) | 12.0 | 49.47 | 0.28 | 86.3 |
| Jan.16 (Day 9) | / | / | / | 117.8 |
| Jan.17 (Day 10) | 11.0 | 33.54 | / | 151.8 |
| Jan.18 (Day 11) | / | / | / | 162.2 |
| Jan.19 (Day 12) | 7.0 | 19.64 | 0.62 | 183.1 |
| Jan.20 (Day 13) | 6.4 | 16.14 | / | 203.8 |
| Jan.21 (Day 14) | 6.7 | 14.71 | 0.93 | 232.5 |
| Jan.22 (Day 15) | 5.2 | 11.78 | 0.59 | 253.6 |
| Jan.24 (Day 17) | 5.1 | 7.45 | 0.95 | 278.1 |
| Jan.26 (Day 19) | 4.9 | 7.59 | 0.93 | 239.1 |
| Jan.27 (Day 20) | 5.0 | 5.56 | 0.97 | 226.7 |
| Jan.31 (Day 24)  (antibiotics discontinued) | 4.8 | 1.16 | / | 175.3 |
| Feb.5 (Day 29) (discharged) | / | / | / | / |
